# Molecular detection of *Bartonella* spp. and *Rickettsia* spp. in bat ectoparasites in Brazil

**DOI:** 10.1371/journal.pone.0198629

**Published:** 2018-06-05

**Authors:** Renan Bressianini do Amaral, Elizabete Captivo Lourenço, Kátia Maria Famadas, Amanda Barbosa Garcia, Rosangela Zacarias Machado, Marcos Rogério André

**Affiliations:** 1 Laboratory of Immunoparasitology, Department of Veterinary Pathology, School of Agricultural and Veterinarian Sciences, Universidade Estadual Paulista (UNESP), Jaboticabal, SP, Brazil; 2 Agricultural and Livestock Microbiology Graduation Program, School of Agricultural and Veterinarian Sciences, Universidade Estadual Paulista (UNESP), Jaboticabal, SP, Brazil; 3 Laboratory of Parasite Arthropods, Department of Animal Parasitology, Institute of Veterinary, Universidade Federal Rural do Rio de Janeiro–UFRRJ, Seropédica, RJ, Brasil; University of Minnesota, UNITED STATES

## Abstract

The family Streblidae comprises a monophyletic group of Hippoboscoidea, hematophagous dipterans that parasitize bats. *Bartonella* spp. and *Rickettsia* spp. have been reported in bats sampled in Europe, Africa, Asia, North, Central and South America. However, there are few reports on the *Bartonella* and *Rickettsia* bacteria infecting Hippoboscoidea flies and mites. While Spinturnicidae mites are ectoparasites found only in bats, those belonging to the family Macronyssidae comprise mites that also parasitize other mammal species. This study investigates the occurrence and assesses the phylogenetic positioning of *Bartonella* spp. and *Rickettsia* spp. found in Streblidae flies and Spinturnicidae and Macronyssidae mites collected from bats captured in Brazil. From May 2011 to April 2012 and September 2013 to December 2014, 400 Streblidae flies, 100 Macronyssidaes, and 100 Spinturnicidae mites were collected from bats captured in two sites in northeastern Nova Iguaçu, Rio de Janeiro, southeastern Brazil. Forty (19.8%) out of 202 Streblidae flies were positive for *Bartonella* spp. in qPCR assays based on the *nuoG* gene. Among the flies positive for the bacterium, six (18%) were *Paratrichobius longicrus*, seven (29%) *Strebla guajiro*, two (40%) *Aspidoptera phyllostomatis*, five (11%) *Aspidoptera falcata*, one (10%) *Trichobius anducei*, one (25%) *Megistopoda aranea*, and 18 (32%) *Trichobius joblingi*, and collected from bats of the following species: *Artibeus lituratus*, *Carollia perspicillata*, *Artibeus planirostris*, *Sturnira lilium*, and *Artibeus obscurus*. Six sequences were obtained for *Bartonella* (*nuoG* [n = 2], *gltA* [n = 2], *rpoB* [n = 1], *ribC* = 1]). The phylogenetic analysis based on *gltA* (750pb) gene showed that the *Bartonella* sequences clustered with *Bartonella* genotypes detected in bats and ectoparasites previously sampled in Latin America, including Brazil. Only one sample (0.49%) of the species *Trichobius joblingi* collected from a specimen of *Carollia perspicillata* was positive for *Rickettsia* sp. in cPCR based on the *gltA* gene (401bp). This sequence was clustered with a ‘*Candidatus* Rickettsia andaenae" genotype detected in an *Amblyomma parvum* tick collected from a rodent in the southern region of Brazilian Pantanal. The sampled Macronyssidae and Spinturnicidae mites were negative for *Bartonella* spp. and *Rickettsia* spp. This study demonstrated the first occurrence of *Bartonella* spp. and *Rickettsia* spp. DNA in Streblidae flies collected from bats in Brazil.

## Introduction

The order Chiroptera is the second largest group of mammals in the world, comprising approximately 20% of mammals and more than 1200 species present in all continents, except Antarctica [1]. In Brazil, about 47% of species diversity is found in urban areas [2].

Among all the ectoparasites of bats, Streblidae flies are the ones most frequently reported in the Neotropics. These ectoparasites are often found associated with bat species of the Phyllostomidae and Noctilionidae families [3,4,5]. The family Streblidae comprises a monophyletic group of Hippoboscoidea dipterans [6,7,8], characterized by adenotrophic viviparity, consisting of not only winged but also brachypterous and apterous species that are obligate hematophagous ectoparasites of bats [5,9].

The mites of the Suborder Mesostigmata belong to four families: Macronyssidae, Laelapidae, Spelaeorhynchidae, and Spinturnicidae. The species belonging to the families Macronyssidae and Laelapidae can parasitize several mammals species, including bats, whereas those of the families Spelaeorhynchidae and Spinturnicidae are known to parasitize Chiroptera exclusively [10,11].

*Bartonella* species includes Gram-negative facultative intracellular alpha-proteobacteria belonging to the order Rhizobiales [12]. These reemerging agents parasitize the erythrocytes and endothelial cells of mammals, being associated with diseases in humans and animals [13,14]. *Bartonella* spp. has been reported in Hippoboscoidea flies collected from bats sampled in the United Kingdom [15], Kenya [16], Taiwan [17], Peru [18], Nigeria [19], Puerto Rico [20], Finland [21], Madagascar [22], Costa Rica [23], Guatemala [24,25], French Guiana [26], Gana [27], Algeria [28], South Africa [29], and more recently, in Brazil [30] and México [31]. For instance, *Bartonella* sp. has been detected in Nycteribiidae flies in Ghana and Slovenia [10], Nigeria [18], Madagascar [22], Costa Rica [23] and Algeria [28]. On the other hand, bartonellae have been detected in Streblidae flies in the USA [32], Puerto Rico, Panama, China, Philippines, Dominican Republic, French Guyana, Mexico, Peru [12], Costa Rica [23], South Africa and Swaziland [29]. Additionally, the role of bats as carriers of *Bartonella* species and genotypes with zoonotic potential has been investigated. For instance, “*Candidatus* Bartonella mayotimonensis”, an agent associated with cases of endocarditis in humans in Iowa, USA [33], has been detected in bats in Finland [34], France, Spain [35], USA [36], and in ectoparasites (flies and fleas) in Finland [33].

The genus *Ricketts*ia includes obligatory intracellular Gram-negative bacteria belonging to the Phylum Proteobacteria, Class Alphaproteobacteria, Order Rickettsiales and Family Rickettsiaceae. The pathogenic *Rickettsia* species, which causes the group of diseases known as rickettsioses, are divided into two groups: Typhus, which comprises species mainly transmitted by fleas, and the Spotted Fever, that include *Rickettsia* species transmitted mostly by ticks [37]. *Rickettsia* spp. has been already detected in bats sampled in the United States [38], Saint Kitts islands, Galapagos [39], South Africa, Swaziland [29], and Argentina [40]. In Brazil, serological evidence of exposure to *Rickettsia* spp. (9.5% to *R*. *rickettsii*, 9.5% to *R*. *parkeri*, 7.8% to *R*. *amblyommii*, and 1.1% to *R*. *rhipicephali*) has been reported among bats sampled in São Paulo state [41].

Although *Bartonella* spp. and *Rickettsia* spp. have not been detected in Macronyssidae and Spinturnicidae mites parasitizing bats so far, these agents were detected in *Ornithonyssus bacoti*, a Macronyssidae mite species found parasitizing rodents in Egypt [42].

Furthermore, *Rickettsia* spp. has been molecularly detected in ticks collected from bats in the United States [38,43], France [44], French Guiana [45], and Poland [46]. Additionally, *Ricketts*ia sp. of the Spotted Fever Group was detected in flies collected from bats sampled in the USA [47] and Malaysia [48].

The present study used molecular techniques to detect and characterize the occurrence of *Bartonella* spp. and *Rickettsia* spp. in flies of the family Streblidae collected from bats sampled in Rio de Janeiro state, Brazil.

## Material and methods

### Study area, sampled animals, and ectoparasites

The bat ectoparasites were collected under license from SISBIO/ICMBio (Sistema de Autorização e Informação em Biodiversidade/Instituto Chico Mendes de Conservação da Biodiversidade), protocol number #28064–2.

The bats were captured during 36 nights from May 2011 to April 2012 and from September 2013 to December 2014 using mist nets (12 × 3 m and 20 mm mesh). The sampling sites were the Tinguá Biological Reserve (22°34′57.4″S; 043°26′15.9″W) and two surrounding areas (22°35′16.53″S; 043°24′13.86″W and 22°36′50.69″S; 043°24′47.17″W) in northeastern Nova Iguaçu, Rio de Janeiro, Brazil. The bats were identified based on Gardner and Dias [49] and Peracchi [50]. Four hundred flies, plus 100 Spinturnicidae and 100 Macronyssidae mites were removed from the bats using forceps and stored in microtubes containing 100% ethanol. The bat flies were identified using a stereoscopic microscope, dichotomous keys and descriptions [51–57]. The nomenclature followed Dick and Graciolli [58] for Streblidae and Gardner [49] for bats, except for *Dermanura*, which has been elevated to generic status [59,60]. The mites were identified in a light microscope, using previously described identification keys [61–63]. The bats were released after sampling.

In total, 400 Streblidae flies were collected: *Paratrichobius longicrus* (n = 49), *Megistopoda aranea* (n = 4), *Aspidoptera phyllostomatis* (n = 8), *Trichobius joblingi* (n = 110), *Trichubius anducei* (n = 10), *Strebla guajiro* (n = 29), *Megistopoda proxima* (n = 77), *Aspidoptera falcata* (n = 107), *Trichobius furmani* (n = 4), and *Strebla wiedemanni* (n = 2). Additionally, 100 Macronyssidae mites of the species *Chiroptonyssus haematophagus*, 100 Spinturnicidae mites of the species *Periglischrus ojasti* (n = 50) and *Periglischrus iheringi* (n = 50) were also collected from bats.

### DNA extraction and quality assessment

DNA was extracted individually from each fly specimen and from pools comprising 10 mites of the Spintunicidae and Macronyssidae specimens, grouped according the species and host from where they were collected, using the Illustra Tissue and Cells Genomic Prep Mini Spin Kit (GE Healthcare Life Sciences), following manufacturer’s instructions. Purified DNA samples were eluted in 100μL. The DNA quality was evaluated by concentration and 260/280 and 260/230 nm absorbance ratios using a spectrophotometer (Nanodrop, Thermo Scientific, USA). Also, a conventional PCR (cPCR) assay, based on a 710-bp fragment of *cox-1* gene [64], was performed to evaluate the absence of inhibitors in DNA samples and the positive samples were submitted to additional *Bartonella* spp. and *Rickettsia* spp. PCR assays. Conventional cPCR assays were performed in a T100™ Thermal Cycler (BioRad™, CA, USA).

### *Bartonella* detection and characterization

A previously described quantitative PCR (qPCR) protocol based on *nuoG* gene [65] was used to detect and quantify *Bartonella* spp. DNA copies (number of copies/μL) in bat biological samples. The qPCR assays were performed in 10 μL final volume reaction mixtures, containing 1 μL of DNA sample, 1.2 μM of each primer F-Bart (5'-CAATCTTCTTTTGCTTCACC-3'), R-Bart (5'- TCAGGGCTTTATGTGAATAC-3') and hydrolysis probe TexasRed-5'-TTYGTCATTTGAACACG-3'[BHQ2a-Q]-3', Master Mix 2x buffer (GoTaq™ Probe qPCR Master Mix, Promega Corporation, Madison, USA) and ultra-pure sterilized water (Nuclease-Free Water, Promega Corporation, Madison, USA) q.s.p. 10 μL. The amplification conditions were 95°C for 3 minutes followed by 40 cycles at 95°C for 10 seconds and 52.8°C for 30 seconds [65]. PCR amplifications were conducted in low-profile multiplate unskirted PCR plates (BioRad™, CA, USA), using a CFX96 Thermal Cycler (BioRad™, CA, USA). Standard curves were constructed with serial dilutions of plasmid DNA (pIDTSMART—Integrated DNA Technologies) (1.0x10⁷ to 1.0x10⁰ copies/μL), which encoded an 83bp *Bartonella henselae-nuoG* gene fragment. The number of plasmid copies was determined by (Xg/μL DNA/ [plasmid length in bp x 660]) x 6.022 x10^23^ x plasmid copies/μL.

All DNA samples were initially tested in duplicates. All duplicates whose Cq difference was higher than 0.5 were re-tested in triplicate. Amplification efficiency (E) was calculated from the slope of the standard curve in each run using the following formula (E = 10^−1/slope^). The standard curves generated by 10-fold dilutions were used to determine the amount of DNA that could be detected with 95% of sensitivity [66].

To perform the molecular characterization of *Bartonella* spp., the qPCR-positive samples were submitted to previously described cPCR assays targeting eight different genic regions, namely *nuoG* (400bp) [67], *ribC* (420bp) [68], *gltA* (750bp) [69], *rpoB* (800bp) [70], the intergenic spacer region 16S-23SrRNA ITS (453-717bp) [71], *groEL* (752bp) [71,72], *fstZ* (600bp) [71], and *pap-31* (564bp) [73]. *Bartonella* sp. previously detected in a specimen of *Sturnira lilium* bat sampled in southern Brazil [30] and sterilized ultrapure water (Nuclease-Free Water, Promega™, Madison, Wisconsin, USA) were used as positive and negative controls, respectively.

### *Rickettsia* detection and characterization

All DNA samples were submitted to a cPCR assay targeting citrate synthase protein-coding gene (*gltA)* (401 bp) to detect and characterize *Rickettsia* spp. [74]. All the positive samples were submitted to cPCR assays targeting the *ompA* (530bp) [75], *ompB* (862 bp) [76] and *htrA* 17-kDa (440bp) [77] genes. The mixture contained 10X PCR buffer (Life Technologies^®^, Carlsbad, CA, USA), 1.0 mM MgCl_2_ (Life Technologies^®^, Carlsbad, CA, USA), 0.2 mM deoxynucleotide triphosphate (dNTPs) mixture (Life Technologies^®^, Carlsbad, CA, USA), 1.5 U Taq DNA Polymerase (Life Technologies^®^, Carlsbad, CA, USA), and 0.5 μM of each primer (Integrated DNA Technologies^®^, Coralville, IA, USA). *Rickettsia rickettsii* DNA, kindly provided by Fundação Oswaldo Cruz (Fiocruz, Rio de Janeiro, Brazil), and ultra-pure sterile water (Life Technologies^®^, Carlsbad, CA, USA) were used as positive and negative controls, respectively.

The products of all cPCR assays were separated by electrophoresis on a 1% agarose gel stained with ethidium bromide (Life Technologies™, Carlsbad, CA, USA) under 100V/150mA for 50 minutes. The gels were imaged under ultraviolet light (ChemiDoc MP Imaging System, Bio Rad™) using the Image Lab Software Version 4.1.

### Sequencing and phylogenetic analyses

Amplified products were purified using the Silica Bead DNA gel extraction kit (Thermo Fisher Scientific™, Waltham, MA, USA) and sequenced using the BigDye™ Terminator v3.1 Cycle Sequencing Kit (Thermo Fisher Scientific™, Waltham, MA, USA) and the ABI PRISM 310DNA Analyzer (Applied Biosystems™, Foster City, CA, USA) [78]. The primers used in the sequencing reactions have been previously described in PCR assays for *Bartonella* spp.

The sequences obtained from positive samples were first submitted to a screening test using Phred-Phrap software version 23 [79,80] to evaluate the electropherogram quality and to obtain consensus sequences from the alignment of the sense and antisense sequences. The BLAST program [81] was used to analyze the sequences of nucleotides (BLASTn), to browse and compare with sequences from an international database (GenBank) [82]. The consensus sequences obtained in this study and those retrieved from GenBank were aligned using the Clustal/W software [83] via Bioedit v. 7.0.5.3 [84]. Phylogenetic inference was based on Bayesian Inference (BI) and Maximum Likelihood (ML) methods. The Bayesian inference (BI) analysis was performed with MrBayes 3.1.2 [85] via CIPRES Science Gateway [86]. Markov Chain Monte Carlo (MCMC) simulations were run for 10⁶ generations with a sampling frequency of every 100 generations and a burn-in of 25%. The Maximum-likelihood (ML) analysis was inferred with the W-IQ-Tree tool available online (http://iqtree.cibiv.univie.ac.at/) [87,88] using 1000 bootstrapping replicates. The best evolution model was selected by the program jModelTest2 (version 2.1.6) on XSEDE [89], under the Akaike Information Criterion (AIC) and Bayesian Information Criterion (BIC) [90]. All trees were examined in Treegraph 2.0.56–381 beta [91].

## Results

### *Bartonella* and *Rickettsia* prevalence in ectoparasites

All mite pools and 202 out of 400 of the Streblidae flies were positive in cPCR assays targeting *cox-1* invertebrate endogenous gene. Forty (19.8%) out of 202 Streblidae flies were positive for *Bartonella* spp. in qPCR assays based on the *nuoG* gene. Among the positive flies, 18% (6/32) were *Paratrichobius longicrus*, 29% (7/24) *Strebla guajiro*, 40% (2/5) *Aspidoptera phyllostomatis*, 11% (5/43) *Aspidoptera falcata*, 10% (1/10) *Trichobius anducei*, 25% (1/4) *Megistopoda aranea*, 32% (18/55) *Trichobius joblingi* and 0% (0/29) *Megistopoda proxima*. The positive flies were collected from bats of the following species: two *Artibeus fimbriatus*, six *Artibeus lituratus*, 26 *Carollia perspicillata*, five *Sturnira lilium*, one *Artibeus obscurus* and one *Artibeus planirostris*. The efficiency, R^2^, slope, and Y-intercept of qPCR assays ranged from 90.5% to 104.7% (mean = 96.32%), 0.987 to 0.998 (mean = 0.986), -3.577 to -3.215 (mean = -3.422), and 36.506 to 39.454 (mean = 38.218), respectively. The quantification of *nuoG Bartonella* spp. ranged from 5.05 x 10^−1^ to 6.08 x 10^4^ copies/μL (Table 1).

**Table 1:** The parameters obtained for Streblidae flies positive for *Bartonella* spp. in qPCR assays based on *nuoG* gene, in Rio de Janeiro state.

Streblidae species	Host	Mean quantification(copies/μL)	E	R^2^	Slope	y-intercept
*Aspidoptera phyllostomatis*	*Artibeus fimbriatus*	4.19 X 10^0^	93.4%	0.984	-3.492	37.176
*Aspidoptera phyllostomatis*	*Artibeus fimbriatus*	6.00 X 10^0^	93.4%	0.984	-3.492	37.176
*Aspidoptera falcata*	*Sturnira lilium*	4.01 X 10^1^	99.4%	0.953	-3.335	39.454
*Aspidoptera falcata*	*Sturnira lilium*	1.00 X 10^−1^	104.8%	0.983	-3.211	35.813
*Aspidoptera falcata*	*Sturnira lilium*	1.93 X 10^2^	95%	0.992	-3.423	37.662
*Aspidoptera falcata* *	*Sturnira lilium*	3.871 X 10^1^; 8.223 X 10^1^	101.6%	0.997	-3.284	35.821
*Aspidoptera falcata* *	*Sturnira lilium*	1.571 X 10^1^; 3.445 X 10^1^	95%	0.992	-3.423	37.662
*Megistopoda aranea*	*Artibeus obscurus*	6.27 X 10^1^	99.4%	0.953	-3.335	39.454
*Paratrichobius longicrus*	*Artibeus lituratus*	3.58 X 10^0^	93.4%	0.984	-3.492	37.176
*Paratrichobius longicrus*	*Artibeus lituratus*	9.23 X 10^0^	90.7%	0.998	-3.566	38.552
*Paratrichobius longicrus*	*Artibeus lituratus*	4.81 X 10^1^	90.7%	0.998	-3.566	38.552
*Paratrichobius longicrus*	*Artibeus lituratus*	6.21 X 10^−1^	104.8%	0.983	-3.211	35.813
*Paratrichobius longicrus* *	*Artibeus lituratus*	2.833 X 10^1^; 1.268 X 10^1^	93.4%	0.984	-3.492	37.176
*Paratrichobius longicrus*	*Artibeus lituratus*	3.58 X 10^0^	93.4%	0.984	-3.492	37.176
*Strebla guajiro*	*Carollia perspicillata*	1.72 X 10^1^	99.4%	0.953	-3.335	39.454
*Strebla guajiro*	*Carollia perspicillata*	7.97 X 10^3^	99.4%	0.953	-3.335	39.454
*Strebla guajiro*	*Carollia perspicillata*	2.27 X 10^0^	101.6%	0.997	-3.284	35.821
*Strebla guajiro*	*Carollia perspicillata*	1.34 X 10^1^	101.6%	0.997	-3.284	35.821
*Strebla guajiro*	*Carollia perspicillata*	8.71 X 10^1^	95%	0.992	-3.423	37.662
*Strebla guajiro*	*Carollia perspicillata*	4.94 X 10^1^	90.7%	0.998	-3.566	38.552
*Strebla guajiro* *	*Carollia perspicillata*	7.535 X 10^0^; 2.923 X 10^0^	99.4%	0.953	-3.335	39.454
*Trichobius joblingi*	*Carollia perspicillata*	2.26 X 10^1^	93.4%	0.984	-3.492	37.176
*Trichobius joblingi*	*Carollia perspicillata*	6.47 X 10^0^	104.8%	0.983	-3.211	35.813
*Trichobius joblingi*	*Carollia perspicillata*	1.05 X 10^−1^	104.8%	0.983	-3.211	35.813
*Trichobius joblingi*	*Carollia perspicillata*	2.23 X 10^1^	101.6%	0.997	-3.284	35.821
*Trichobius joblingi*	*Carollia perspicillata*	5.62 X 10^0^	95%	0.992	-3.423	37.662
*Trichobius joblingi*	*Carollia perspicillata*	2.80 X 10^0^	90.7%	0.998	-3.566	38.552
*Trichobius joblingi*	*Carollia perspicillata*	6.06 X 10^0^	90.7%	0.998	-3.566	38.552
*Trichobius joblingi*	*Carollia perspicillata*	9.56 X 10^0^	90.7%	0.998	-3.566	38.552
*Trichobius joblingi*	*Carollia perspicillata*	2.46 X 10^0^	90.7%	0.998	-3.566	38.552
*Trichobius joblingi* *	*Carollia perspicillata*	1.703 X 10^0^; 2.383 X 10^0^	93.4%	0.984	-3.492	37.176
*Trichobius joblingi* *	*Carollia perspicillata*	3.45 X 10^−1^; 1.58 X 10^0^	104.8%	0.983	-3.211	35.813
*Trichobius joblingi* *	*Carollia perspicillata*	5.073 X 10^−1^; 1.753 X 10^0^	104.8%	0.983	-3.211	35.813
*Trichobius joblingi*	*Carollia perspicillata*	3.355 X 10^−1^; 6.424 X 10^−1^	104.8%	0.983	-3.211	35.813
*Trichobius joblingi*	*Carollia perspicillata*	5.25 X 10^1^	99.4%	0.953	-3.335	39.454
*Trichobius joblingi* *	*Carollia perspicillata*	2.141 X 10^−1^; 7.950 X 10^−1^	101.6%	0.997	-3.284	35.821
*Trichobius joblingi*	*Carollia perspicillata*	6.50 X 10^0^	90.7%	0.998	-3.566	38.552
*Trichobius joblingi* *	*Carollia perspicillata*	1.148 X 10^0^; 1.987 X 10^0^	95%	0.992	-3.423	37.662
*Trichobius anducei* *	*Carollia perspicillata*	2.922 X 10^0^; 6.016 X 10^0^	101.6%	0.997	-3.284	35.821

E = Efficiency of qPCR assays; R^2^ = determination coefficient

*Samples marked with "*" show the result for each replicate rather than the parameter average. This is due to the low DNA concentrations in these samples, which generated differences in Cq values of the replicates higher than 0.5 (Monte Carlo effect–[66]).

Ten (25%) out of 40 positive samples in the qPCR were also positive for at least one target gene in cPCR assays for *Bartonella* spp., including 6 (15%) for the *nuoG gene*, 2 (5%) for the *gltA* gene, 4 (10%) for the *ribC* gene, 3 (7,5%) for the *groEL* gene, 1 (5%) for the *ftsZ* gene, and 1(2,5%) for the *rpoB* gene. None was positive for *pap-31* and for the intergenic spacer 16S-23S rRNA (ITS) (Table 2). Only six *Bartonella* spp. sequences were obtained (*nuoG* [n = 2], *gltA* [n = 2], *rpoB* [n = 1], *ribC* [= 1]) due to low intensity of some amplified products, which precluded high quality sequencing. The sequences obtained were deposited to the GenBank under accession numbers MG551538, MG654770-MG654774 (Table 3).

**Table 2:** Streblida flies positive for *Bartonella* spp. in both qPCR and cPCR assays targeting different genes.

Streblidae species	Host	qPCR Mean quantification(*nuoG* copies/μL)	cPCR							
			*gltA*	*rpoB*	*nuoG*	*groEL*	*ribC*	*ftsZ*	*pap-31*	ITS
*Strebla guajiro*	*Carollia perspicillata*	7,97 X 10^3^	Seq	Seq	Seq	NS	Seq	NS	_	_
*Paratrichobius longicrus*	*Artibeus lituratus*	3,58 X 10^0^	_	_	NS	_	_	_	_	_
*Paratrichobius longicrus*	*Artibeus lituratus*	4,81 X 10^1^	_	_	NS	_	_	_	_	_
*Megistopoda aranea*	*Artibeus obscurus*	6,27 X 10^1^	_	_	Seq	_	_	_	_	_
*Aspidoptera falcata*	*Sturnira lilium*	4,01 X 10^1^	_	_	NS	_	_	_	_	_
*Trichobius joblingi*	*Carollia perspicillata*	2,23 X 10^1^	_	_	NS	_	_	_	_	_
*Aspidoptera falcata*	*Sturnira lilium*	1,93 X 10^2^	_	_	_	NS	NS	_	_	_
*Aspidoptera phyllostomatis*	*Artibeus fimbriatus*	6,00 X 10^0^	_	_	_	NS	_	_	_	_
*Trichobius joblingi*	*Carollia perspicillata*	2,26 X 10^1^	_	_	_	_	NS	_	_	_
*Strebla guajiro*	*Carollia perspicillata*	1,72 X 10^1^	Seq	_	_	_	NS	_	_	_

ITS = intergenic transcriber spacer; NS = positive sample in cPCR but not sequenced due to the low intensity of amplified products; Seq = Sequences obtained and deposited in the GenBank database.

**Table 3:** Maximum identity by Blast analysis of *Bartonella* and *Rickettsia* sequences detected in Streblidae flies collected from bats sampled in Rio de Janeiro state, Brazil.

GenBank accession number	Bat fly species	Host	Target gene	Query coverage	Closest GenBank Match
MG551538	*Strebla guajiro*	*Carollia perspicillata*	*gltA*	99%	98% Uncultured *Bartonella* sp. clone SJ112 (KJ816687)
MG65470	*Strebla guajiro*	*Carollia perspicillata*	*gltA*	88%	93% Uncultured *Bartonella* sp. clone SJ118 (KJ816665)
MG65471	*Strebla guajiro*	*Carollia perspicillata*	*nuoG*	100%	93% *Bartonella alsatica* (EF659935)
MG65472	*Megistopoda aranea*	*Artibeus obscurus*	*nuoG*	100%	93% *Bartonella sp*. WD16.2 (CP019781)
MG65473	*Strebla guajiro*	*Carollia perspicillata*	*ribC*	98%	84% *Bartonella washoensis* (AB292599)
MG65474	*Strebla guajiro*	*Carollia perspicillata*	*rpoB*	100%	89% *Bartonella sp*. Khabarovsk-17 (AB779537)
MG65475	*Trichobius joblingi*	*Carollia perspicillata*	*gltA*	100%	100% ‘*Candidatus* Rickettsia andeanae’ (KT153033)

In PCR assays, one (0.49%) out of 202 flies samples was positive for *Rickettsi*a spp. based on the *gltA* gene, being identified as ‘*Candidatus* Rickettsia andeanae’ after sequencing (Table 3). However, because this positive sample had low amount of rickettsial DNA, the subsequent PCR assays based on the *ompA*, *ompB* and *htrA* 17-kDa genes were negative, precluding additional phylogenetic inferences.

The 100 Macronyssidae (*Chiroptonyssus hematophagous* collected from *Molossus molossus* and *Molossus rufus)* and 100 Spinturnicidae (*Periglischrus iheringi* [n = 50] collected from *Artibeus lituratus* and *Periglischrus ojasti* (n = 50) collected from *Sturnira lilium*) mites were all negative for both *Bartonella* spp. and *Rickettsia* spp.

### BLAST analysis and phylogenetic inference

Based on BLAST analysis, while one *Bartonella nuoG* sequence (GenBank accession number MG65471) was 93% identical to *Bartonella* sp. WD16.2 previously isolated from a deer sampled in Japan (GenBank accession number CP019781), the other GenBank accession number MG65472) was 84% identical to *B*. *alsatica* (GenBank accession number EF659935). Two *Bartonella gltA* sequences GenBank accession numbers MG551538) were 93–98% identical to *Bartonella* sp. detected in bats sampled in Costa Rica (KJ816665 and KJ816687). One *Bartonella rpoB* sequence (GenBank accession number MG65474) was 89% identical to *Bartonella* sp. Khabarovsk detected in Asian mammals (AB779537). Finally, one *Bartonella ribC* sequence (GenBank accession number MG65473) was 84% identical to *B*. *washoensis* (AB292599). The query coverage ranged from 96% to 100% in all BLAST analyses carried out for the *Bartonella* sequences (Table 3).

The only detected *gltA*-*Rickettsia* sp. was 100% identical to '*Candidatus* Rickettsia andeanae' (GenBank accession number MG65475), previously described in a tick sample of the species *Amblyomma parvum*, collected from a rodent found in the Pantanal Sul-Matogrossense, Brazil, with query coverage of 100% (Table 3).

**Fig 1:**
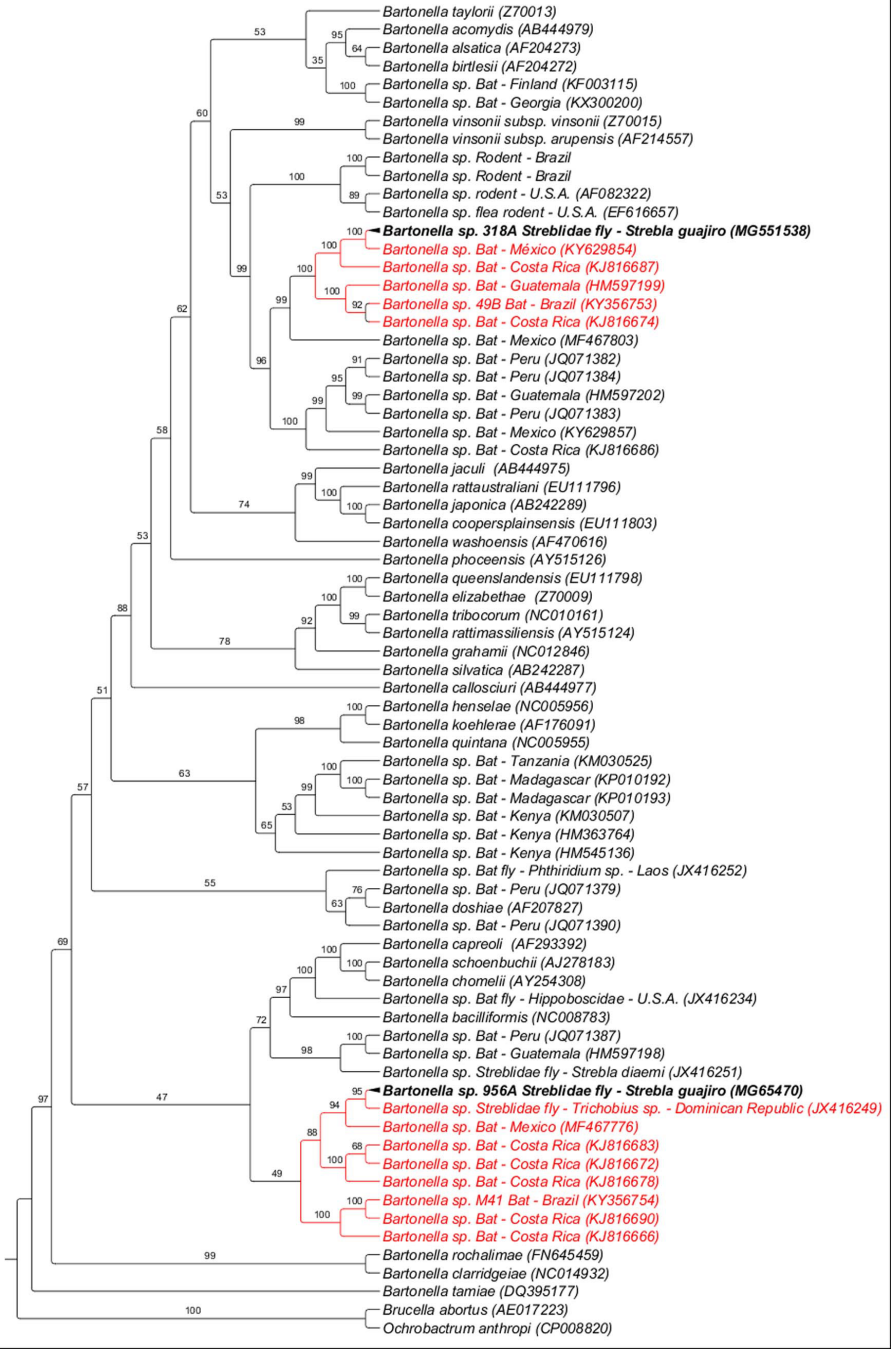
Phylogenetic analysis of *Bartonella gltA* sequences (750 pb) based on the Bayesian Inference method (BI) with the GTR+I+G4 model. The numbers at the nodes correspond to bootstrap values accessed with 1,000 replicates. *Brucella abortus* and *Ochrobactrum anthropi* were used as outgroups.

The phylogenetic tree inferred by Bayesian analysis based on sequences of the *Bartonella gltA* gene formed two distinct clusters. The *Bartonella* sequence (GenBank accession number MG551538) detected in a *Strebla guajiro* specimen collected from *Carollia perspicillata* in Rio de Janeiro state was positioned alone in a branch but closely related to *Bartonella* genotypes previously detected in bats from South America, one genotype detected in a bat (*Sturnira lillium*) in Paraná state, Brazil (KY356753), and other genotypes detected in bats from Guatemala, Mexico, and Costa Rica, with 100% branch support. Additionally, such sequences were positioned in a larger clade related to *Bartonella* sequences detected in rodents sampled in Brazil and U.S.A., together with a *Bartonella* genotype detected in a *Polygenis gwyni* flea collected from a *Sigmodon hispidus* rodent in the U.S.A., with clade support value ​​of 83% in BI analysis. In another cluster, the *Bartonella* sequence (GenBank accession number MG65470) obtained in a *Strebla guajiro* specimen collected from *Carollia perspicillata* sampled in Rio de Janeiro state was closely positioned to a genotype previously detected in a specimen of *Trichobius* sp. fly collected in the Dominican Republic (JX416249), together with genotypes detected in bats in Mexico (MF467776) and Costa Rica (KJ816683; KJ816678; KJ816672), with branch support value ​​of 86% probability in BI analysis. In addition, a larger clade grouped a *Bartonella* genotype (KY356754) detected in a specimen of *Glossophaga soricina* sampled in Paraná, Brazil, and sequences previously detected in cervids and bovines, such as *B*. *capreoli* (AF293392), *B*. *schoenbuchii* (AJ278181) and *B*. *chomelii* (AY254308), with 55% clade support in the BI analysis (Fig 1).

**Fig 2:**
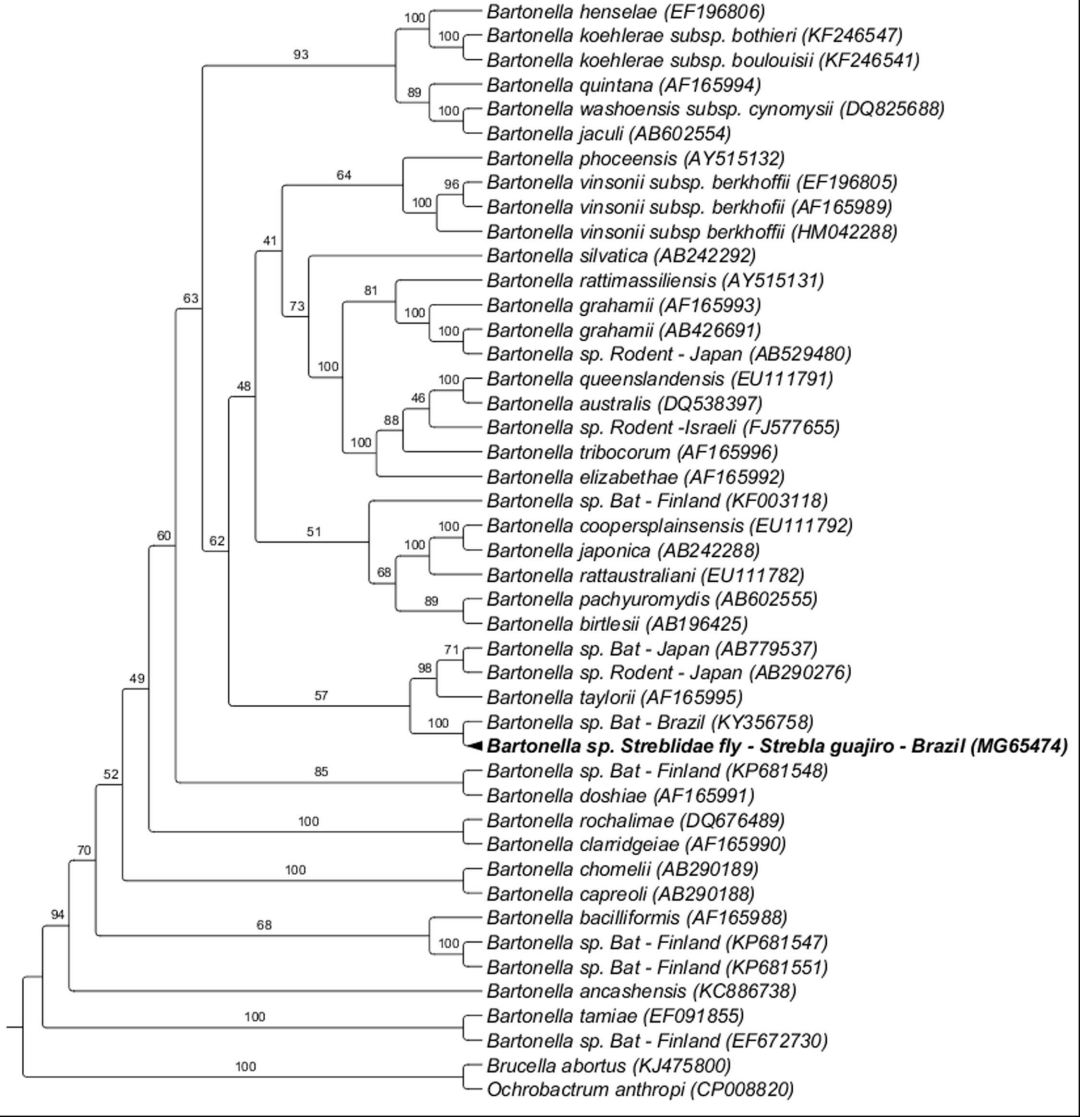
Phylogenetic analysis of *Bartonella rpoB* sequences (800 pb) based on the Bayesian Inference method (BI) with the TPM2u+I+G4 model. The numbers at the nodes correspond to bootstrap values with 1,000 replicates. *Brucella abortus* and *Ochrobactrum anthropi* were used as outgroups.

The *Bartonella rpoB* sequence (GenBank accession number MG65474) obtained from a *M*. *aranea* specimen collected from *C*. *perspicillata* sampled in Rio de Janeiro state was closely related to a genotype detected in a bat (*S*. *lillium*) previously sampled in Paraná state, southern Brazil, with 100% of branch support. These two sequences were positioned in the same cluster formed by *Bartonella taylorii* (AF165995) and *Bartonella* genotypes detected in rodents (AB290276) and in a bat (*Myodes rufocanus*) (AB779537) from Asia, with a branch support of 93% of probability BI analysis (Fig 2).

**Fig 3:**
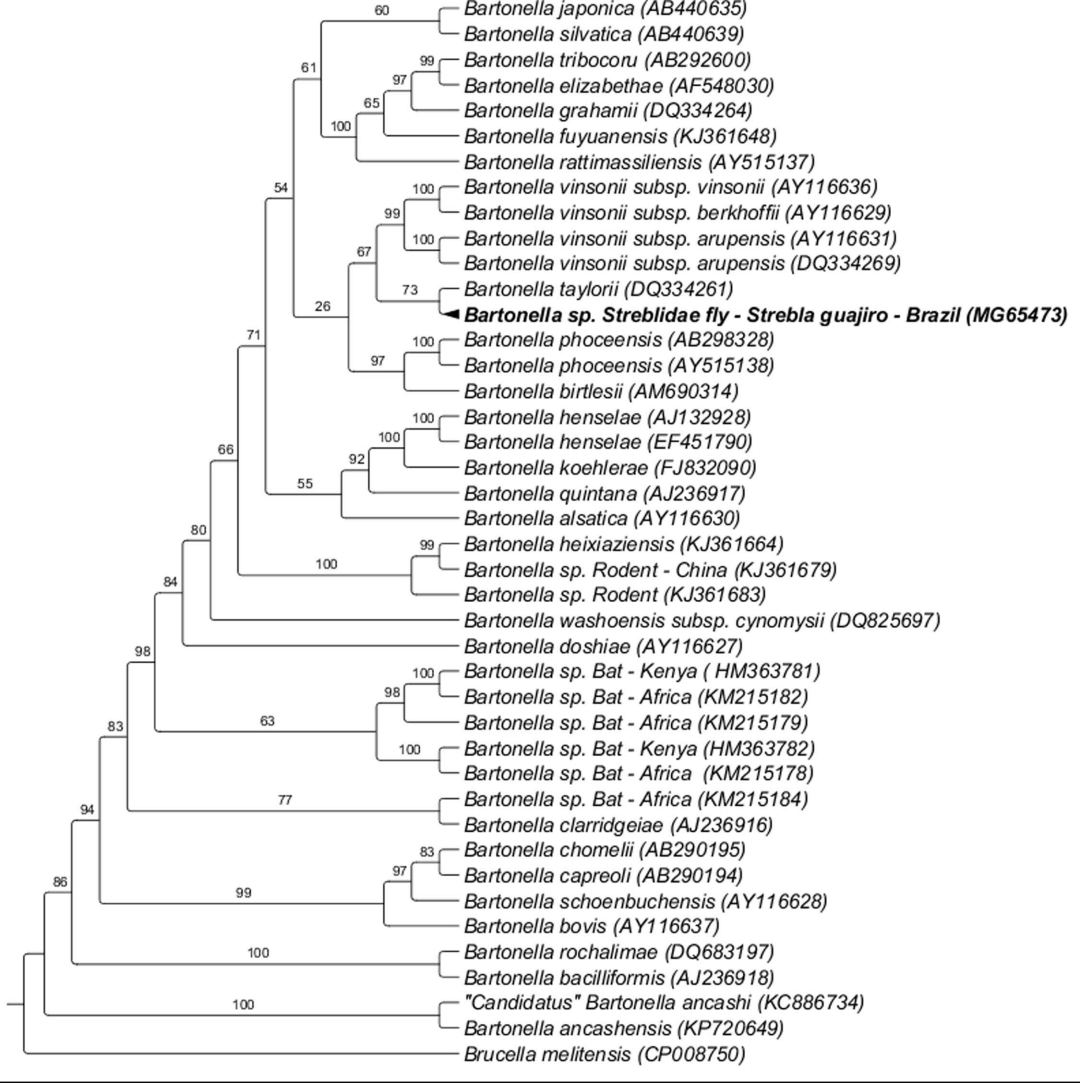
Phylogenetic analysis of *Bartonella ribC* sequences (420 bp) based on the Bayesian Inference method (BI) with the TIM+I+G4 model. The numbers at the nodes correspond to bootstrap values accessed with 1,000 replicates. *Brucella melitensis* were used as outgroups.

The *Bartonella ribC* sequence (GenBank accession number MG65473) obtained from a *M*. *arenea* specimen collected from *C*. *perspicillata* sampled in Rio de Janeiro state was positioned alone in a branch by BI analysis, but closely related (74% of branch support) to *Bartonella tribocorum* (AB292600), *Bartonella elizabethae* (AF548030), *Bartonella grahamii* (DQ334264), *Bartonella fuyuanensis* (KJ361648), and *Bartonella rattimassiliensis* (AY515137) (Fig 3).

**Fig 4:**
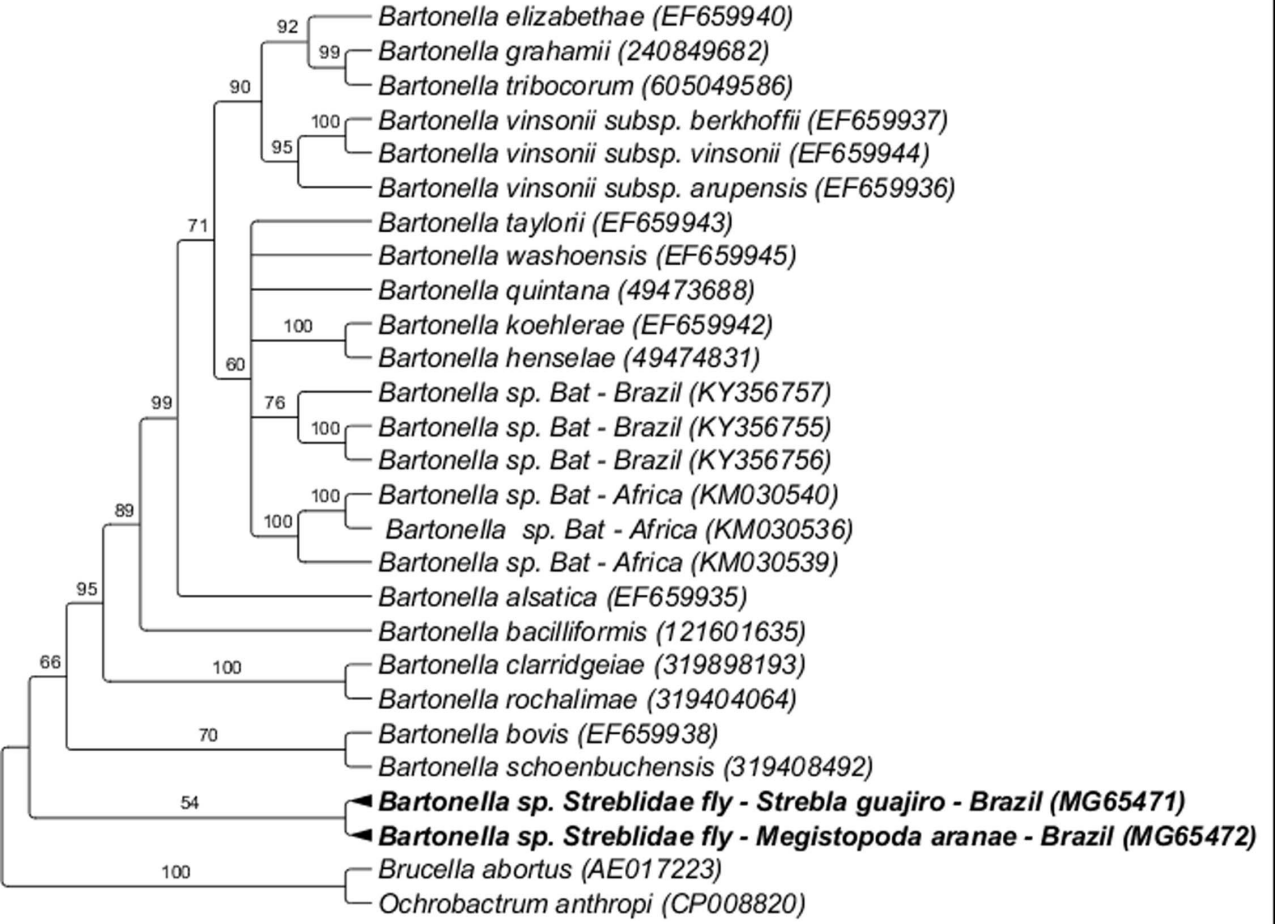
Phylogenetic analysis of *Bartonella nuoG* sequences (400 pb) based on the Bayesian Inference method (BI) with the GTR+I+G4 model. The numbers at the nodes correspond to bootstrap values accessed with 1,000 replicates. *Brucella abortus* and *Ochrobactrum anthropi* were used as outgroups.

The *Bartonella* nuoG sequences (GenBank accession numbers MG65471; MG65472) obtained from *Strebla guajiro* and *Megistopoda aranea* specimens collected from bats of the species *Carollia perspicillata* and *Artibeus obscuros*, respectively, in Rio de Janeiro state, were positioned in a single clade separated from the others described, with 92% clade support in BI analysis (Fig 4).

**Fig 5:**
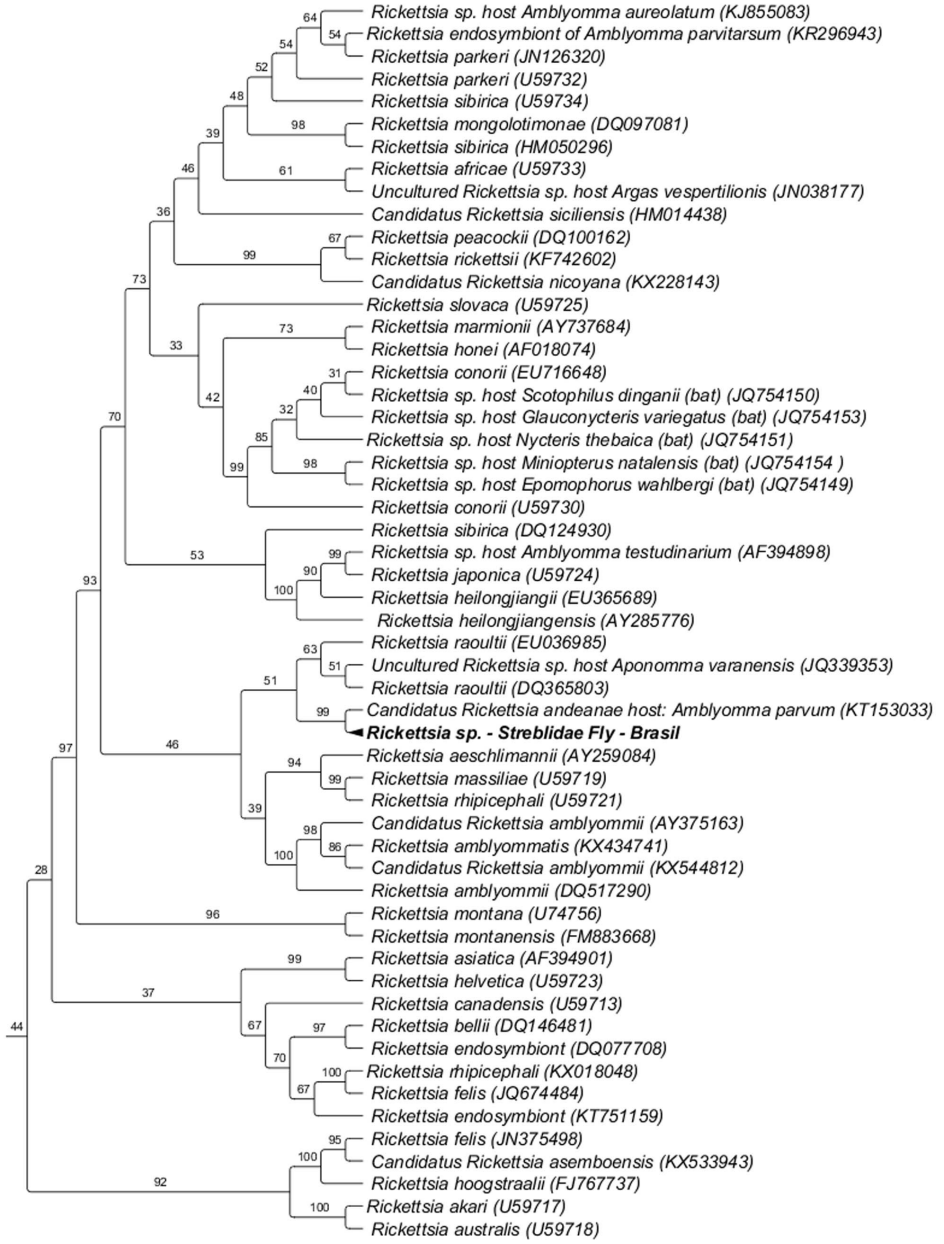
Phylogenetic analysis of *Rickettsia gltA* sequences (401 bp) based on the Maximum Likelihood (ML) method with the TIM+I+G4 model. The numbers at the nodes correspond to bootstrap values accessed with 1,000 replicates. *Rickettsia prowazekii* were used as outgroups.

The *Rickettsia gltA* sequence obtained in a specimen of *Trichobius joblingi* collected from *C*. *perspicillata* sampled in Rio de Janeiro was closely related to a ‘*Candidatus* Rickettsia andeanae’ previously detected in *A*. *parvum* tick collected from a rodent in the wetlands of Pantanal, Brazil, with 99% with branch support of in ML analysis (Fig 5).

## Discussion

Studies on bats and diseases caused by bacteria have increased worldwide due to the role of this mammal group as reservoirs, hosts, and sources of infection of several pathogens [92]. The present work reports the occurrence and molecular characterization of *Bartonella* spp. and *Rickettsia* spp. in Streblidae flies parasites of bats in two localities in Rio de Janeiro state, southeastern Brazil. Streblidae flies are strictly hematophagous ectoparasites of bats, with usually high specificity for hosts. The Streblidae fly species usually parasites a single bat species or some closely related species [3,93,94], such parasitism pattern was also observed in the present study, in which a certain Streblidae species was found parasitizing no more than two different bat species belonging to the same genus or family.

Furthermore, *Bartonella* occurrence was lower in Streblidae flies (19.8% [41/202]) compared to the 66.4% (91/137) in Nycteribiidae flies collected from bats in Ghana [94], 41.7% (10/34) in Nigeria [18], and 72.7% (8/11) in Algeria [27]. Additionally, the prevalence of Streblidae flies collected from bats in this study was lower than that found in Costa Rica 51.8% (29/55) [22] but similar to that found in Nycteribiidae flies in Malaysia 26% (12/42) [48].

In this study, the occurrence of *Rickettsia* spp. was lower in Streblidae flies (0.49%) compared to *Trichobius major* flies collected in bats in the USA (1.16%) [47], and *Eucampsipoda madagascarensis* (5.5%) and *Penicillidia leptothrinax* (15.3%) sampled in Malaysia [48]. In South Africa and Swaziland, all 5 Nycteribiidae flies of the genus *Eucampsipoda* sampled were negative for *Rickettsia* spp. [28]. Nycteribiidae flies sampled in Algeria [27] and Streblidae flies in the islands of Saint Kitts, Galapagos, were negative for *Rickettsia* spp. [40].

Although the real role of Streblidae flies in the transmission of *Bartonella* spp. has not yet been confirmed, previous studies suggest that these dipterans may play an important role as invertebrate hosts for this group of pathogens, harboring a large diversity of *Bartonella* genotypes [11]. In the present study, two different *Bartonella* genotypes were observed in two *Strebla guajiro* specimens of the same Streblidae species, both collected from bats of the species *Carollia perspicillata*, which were placed in different clades in the phylogeny based on the *gltA* gene.

The *Bartonella* spp. was observed especially in Streblide flies collected from bats of the species *Carollia perspicillata* and *Sturnira lilium*, which have been previously recognized as hosts of the new *Bartonella* genotypes in Brazil [30]. However, a previous study with bats in Brazil reported the occurrence of *Bartonella* spp. (5.28%) lower than that found in this study (19.8%). This result corroborates the hypothesis that hemoconcentration occurs in the digestive tract of arthropods, which could improve the molecular diagnosis sensitivity of *Bartonella*. Thus, molecular assays performed on arthropods collected from hosts could reflect a more sensitive epidemiological model [95].

Recent studies with species of Nycteribiidae flies collected from bats in Madagascar aimed to relate bacterial ecology, transmission routes and host-vector specificity [48]. According to Wilkinson et al. [48], certain *Bartonella* genotypes and Nycteribiidae fly species may form mutualistic interactions, which may lead to host specificity. In the aforementioned study, although the found *Bartonella* genotypes were allocated in five different groups, an interchange of *Bartonella* genotypes was observed between *Cyclopodia dubia* and *Basilia* sp., Nycteribiidae flies that did not share the same bat species as hosts. The authors have suggested the existence of direct or indirect mechanisms among the vertebrate hosts that could lead to the intra-specific diversity of *Bartonella* observed in this family of ectoparasites [48]. Similarly, in this study, the phylogeny based on *gltA* gene showed that the *Bartonella* genotype detected in the *Strebla guajiro* specimen collected from *Carollia perspicillata* was closely related to a *Bartonella* genotype detected in a specimen of *Trichobius* sp. collected from *Phyllonycteris poeyi*, a bat species restrictedly distributed in Central America [96]. Therefore, Streblidae flies could act as interchangers of different *Bartonella* genotypes among their vertebrate hosts, leading to intra-specific diversity.

In conclusion, the phylogenetic inference based on *gltA* sequences also demonstrated that one of the *Bartonella* genotypes detected in a *S*. *guajiro* specimen collected from *C*. *perspicillata* was closely related to *Bartonella* genotypes previously detected in bats from Latin America. Additionally, this same *Bartonella* genotype also clustered with sequences previously detected in rodents sampled in the USA [97] and Brazil [98]. Similarly, the phylogenetic inference based on *rpoB* sequences also demonstrated that one *Bartonella* genotype detected in a *S*. *guajiro* specimen collected from *C*. *perspicillata* was closely related to *Bartonella* genotypes previously detected in bats from Brazil and Japan, and to *Bartonella* sp. detected in rodents from Japan. From an evolutionary point of view, this phylogenetic positioning may suggest an association between *Bartonella* genotypes that circulate in rodents and bats, although there are no reports of parasitism by Streblidae flies in rodents [12]. Dietrich et al. [28] reports that *Bartonella* genotypes found in bats in South Africa and Swaziland also clustered with those detected in rodents sampled in Africa, with a low clade support.

The phylogenetic inference based on the *gltA* gene also showed a relationship between *Bartonella* genotypes detected in Strebidae and bats in Latin America with *Bartonella* species found in ruminants. Previously, a *Bartonella* genotype detected in a *Carollia perspicilata* specimen was closely related to a clade containing sequences of *B*. *chomelli* and *B*. *schoenbuchensis*, also isolated from ruminants, in a phylogenetic analysis based on the *ftsZ* gene [30].

Although with low clade support, *Rickettsia* genotypes detected in bats in South Africa and Swaziland were previously grouped with *Rickettsia conorii* [28], the causative agent of Mediterranean spotted fever [99] that has recently been detected in *Rhipicephalus sanguineus* ticks collected from rodents in Nigeria [100]. Similarly, the *Rickettsia* genotype detected in a *Trichobius joblingi* specimen collected from *C*. *perspicillata* in this study was phylogenetically related to the '*Candidatus* Rickettsia andenae' detected in a *Amblyomma parvum* tick found parasitizing a rodent trapped in the Brazilian Pantanal [101]. *A*. *parvum* is a tick species that parasitizes several mammal species during its life cycle while the adult tick parasitizes mainly medium and large mammals (ruminants, equids, and carnivores), the larva and nymph are frequently found in small animals [102]. ‘*Candidatus* Rickettsia andeanae´, whose zoonotic potential remains unknown [103], has been reported infecting ticks in Peru (*Amblyomma maculatum* and *Ixodes bolivensis*), Argentina (*A*. *parvum*) [104], and Paraguay (*A*. *parvum*) [105]. This agent was also detected in *A*. *parvum* collected from horses in the Pantanal biome in Brazil [103], in *A*. *parvum* and *Amblyomma auricularium* collected from horses and *Turdus amaurochalinus* in Northeast Brazil [103], and in *Amblyomma sculptum* collected from a wild animal in Mato Grosso, in central-western Brazil [106; 107]. More recently, '*Candidatus* Rickettsia andeanae' was detected in *A*. *parvum* ticks collected from rodents in the wetlands of Pantanal, Brazil [101].

Although the occurrence of *Bartonella* and *Rickettsia* has not been previously reported in mites of the family Spinturnicidae, *Bartonella* spp. and *Rickettsia* have been molecularly detected in Macronyssidae mites collected from rodents in Egypt [42]. In the aforementioned study, BLAST analysis showed 81% identity with *Bartonella* sp. SE-BartB detected in a flea in Egypt. Regarding *Rickettsia*, the genotypes obtained in the study showed 100% identity with those previously detected in fleas in the U.S. and Egypt [108; 109]. However, the possible role of Macronyssidae and Spinturnicidae mites acting as reservoirs and vectors of *Bartonella* spp. and *Rickettsia* spp. among bats is still unknown.

Even though bat ectoparasites (flies, fleas, and mites) have not been found parasitizing rodents so far, the hypothesis of ticks parasitizing both mammal groups due to their low specificity in relation to their hosts, cannot be ruled out considering the high specificity between these arthropods and bats [3; 4; 93]. *Ornithodoros mimon*, an Argasid tick species described parasitizing bats in South America [110; 111; 112], has already been found in rodents in Brazil [113]. Landulfo et al. [114] simulated the life cycle of this tick species in laboratory conditions, using rabbits and rodents as hosts. The authors found a feeding pattern of *O*. *mimon* larval stage similar to that found in bats, demonstrating that this tick species can parasitize both rodents and bats. In addition, the occurrence of rodent ectoparasites in bats cannot be ruled out. This fact could explain the phylogenetic association between *Bartonella* and *Rickettsia* genotypes found in bats and rodents. The parasitism of bats by immature stages of *A*. *parvum* infected with *'Candidatus* Rickettsia andeanae', for example, could explain the occurrence of this *Rickettsia* species in Streblidae flies. Mutual association between bats and rodents in the same habitat, such as caves, could provide ecological opportunities for exposure and sharing various ectoparasites and pathogens [48].

Finally, it is highlighted that the *Bartonella* genotypes detected in bat ectoparasites in this study were closely related to those previously detected in rodents and bats in Brazil [29; 96]; additionally, the detected *Rickettsia* genotype was shown to be closely related to *'Candidatus* Rickettsia andenae' detected in a tick collected from a rodent in Brazil. In addition, further studies on the vector capacity of Streblidae dipterans in the transmission of *Bartonella* and *Rickettsia* among bats are needed, since 75% of emerging infectious diseases comprises zoonosis, and most of them are transmitted by arthropod vectors [115]. The increase of ecotourism in caves in Brazil associated with the fact that 47% of bat species diversity is found in urban areas [2] emphasize the need of further studies on bacterial zoonotic agents circulating in bats and ectoparasites.

Finally, the results of this study raise an interesting question about the phylogenetic relationship between the *Bartonella* spp. genotypes found in Streblidae flies according to the criteria defining a cut-off point for the *Bartonella* species based on the sequence identity of five gene regions (16S rRNA, *gltA*, *groEL*, *rpoB*, *ftsZ*, *ribC*) and of 16S-23S Intergenic spacer (ITS) previously established by La Scola et al. [116]. The low identity of the sequences with others previously described in GenBank allows suggesting that the genotypes found belong to a new *Bartonella* species circulating in bat ectoparasites, but phylogenetically close to those found in bats, rodents and ruminants.

To the best of authors’ knowledge, the present work presents the first evidence of *Bartonella* and *Rickettsia* DNA in Streblidae flies collected from bats in Brazil. Future studies to evaluate the role of Streblidae flies as vectors for bacterial zoonotic agents in bats are desirable.
